# Supplementary material for: Superfluid response of an atomically thin gate-tuned van der Waals superconductor
Source: Nat Commun. 2023 Apr 12;14:2055. doi: 10.1038/s41467-023-37210-8 (PMC10097715; doi:10.1038/s41467-023-37210-8)
Supplement: Supplementary file 1 — Supplementary Information [file 41467_2023_37210_MOESM1_ESM.pdf]

Supplementary Information for *Superfluid response of an atomically thin gate-tuned van der Waals superconductor*

Alexander Jarjour, G.M. Ferguson, Brian T. Schaefer, Menyoung Lee,

Yen Lee Loh, Nandini Trivedi, Katja C. Nowack

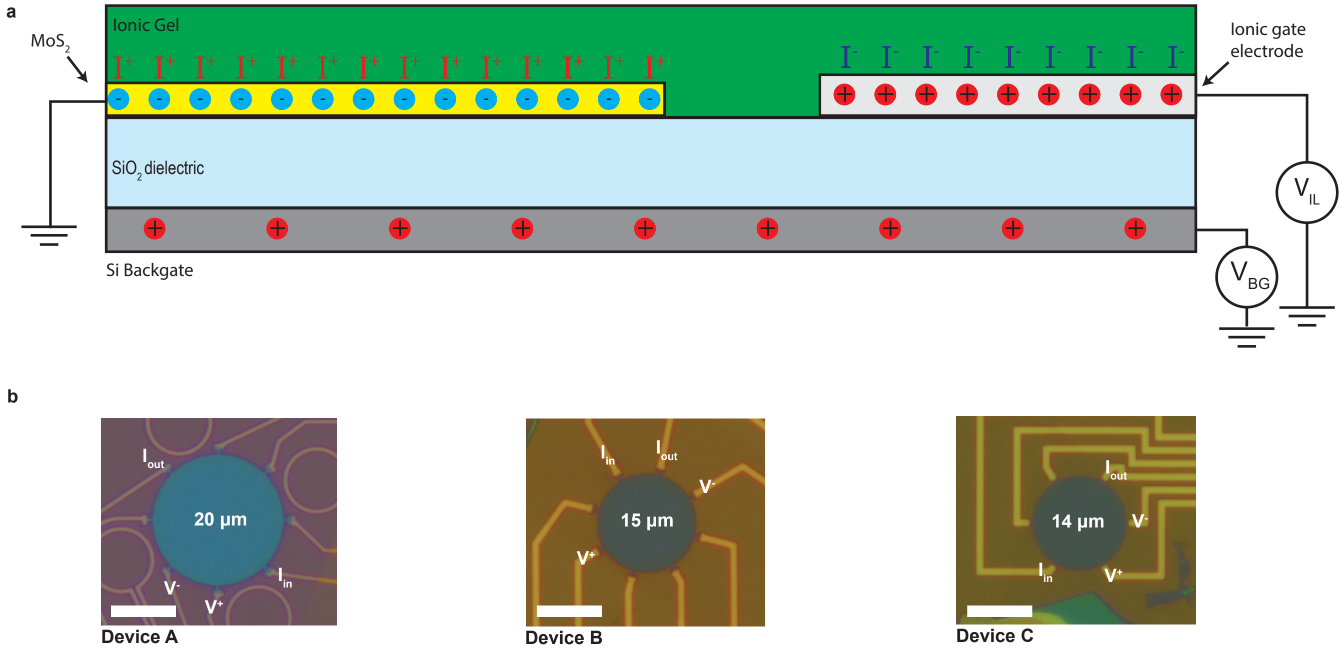

Supplementary Figure 1: Geometry of device and charge distribution. a, Vertical cross-section of a device. Thicknesses and in-plane dimensions are not to scale. The ionic gate electrode is hundreds of microns away from the MoS_2 device. Ions are shown as I^+ and I^-, charge accumulation is shown by colored circles. The backgate is shown at positive voltage, inducing additional electron charge accumulation in the MoS_2 layer. **b**, Optical images of devices A, B, C labels showing the geometry of the four point measurement used to determine the resistance. Diameter of each disk indicated at the center. Device A and B are from the main text, device C is a supplementary device discussed later in this Supplement. All scale bars 10 μm.

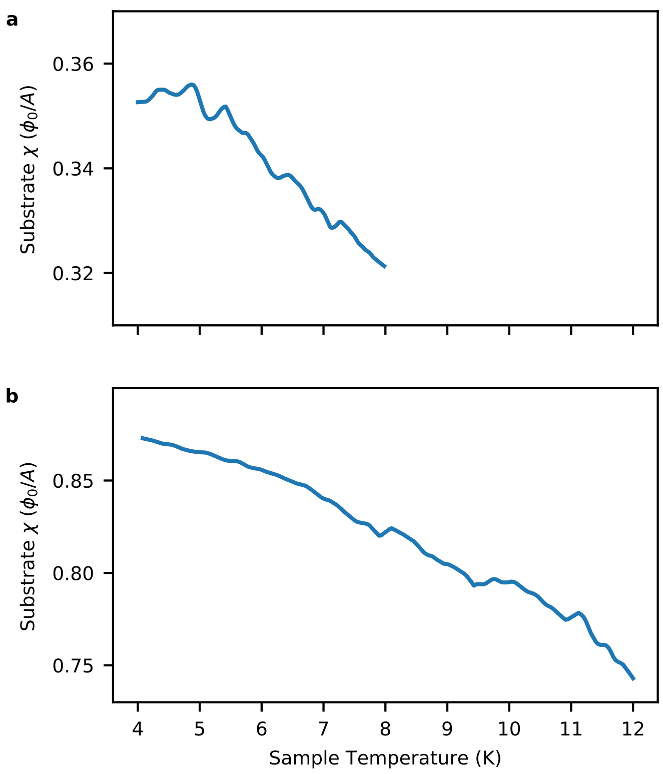

Supplementary Figure 2: Magnetic response of ionic liquid. **a**, Background subtracted from data for device A. **b**, Background subtracted from data for device B.

Supplementary Data: Additional Device

In Supplementary Fig. 3, we present data on an additional device labeled device C, with a diameter of 14 μm. We plot the sheet resistance versus temperature and backgate voltage. Some of the qualitative behavior of device A and B is also observed in device C: the low temperature superfluid response and the sheet resistivity monotonically increase and decrease with increasing backgate voltage, respectively, whereas T_c changes non-monotonically. The sheet resistance is comparable to device A. For device C, we do not have on-chip structures that let us estimate the height of the SQUID (see next section), and the ionic gel was prepared in a different batch than device A and B. A quantitative analysis of the superfluid response is therefore difficult, because the SQUID height cannot be accurately estimated. In addition, the temperature range does not include sufficiently high temperatures to determine the normal state resistivity. We, therefore, omit the comparison of the low temperature superfluid stiffness and the resistivity.

The behavior of the superfluid response in device C close to T_c is distinct from device A and B. In device C the superfluid response initially rises with a low slope, and starts rising sharply at a lower temperature. The onset of the superfluid response does coincide with the point at which the resistivity saturates to a low value. A possible explanation is that this behavior is related to the BKT transition, in the sense that free vortices and antivortices

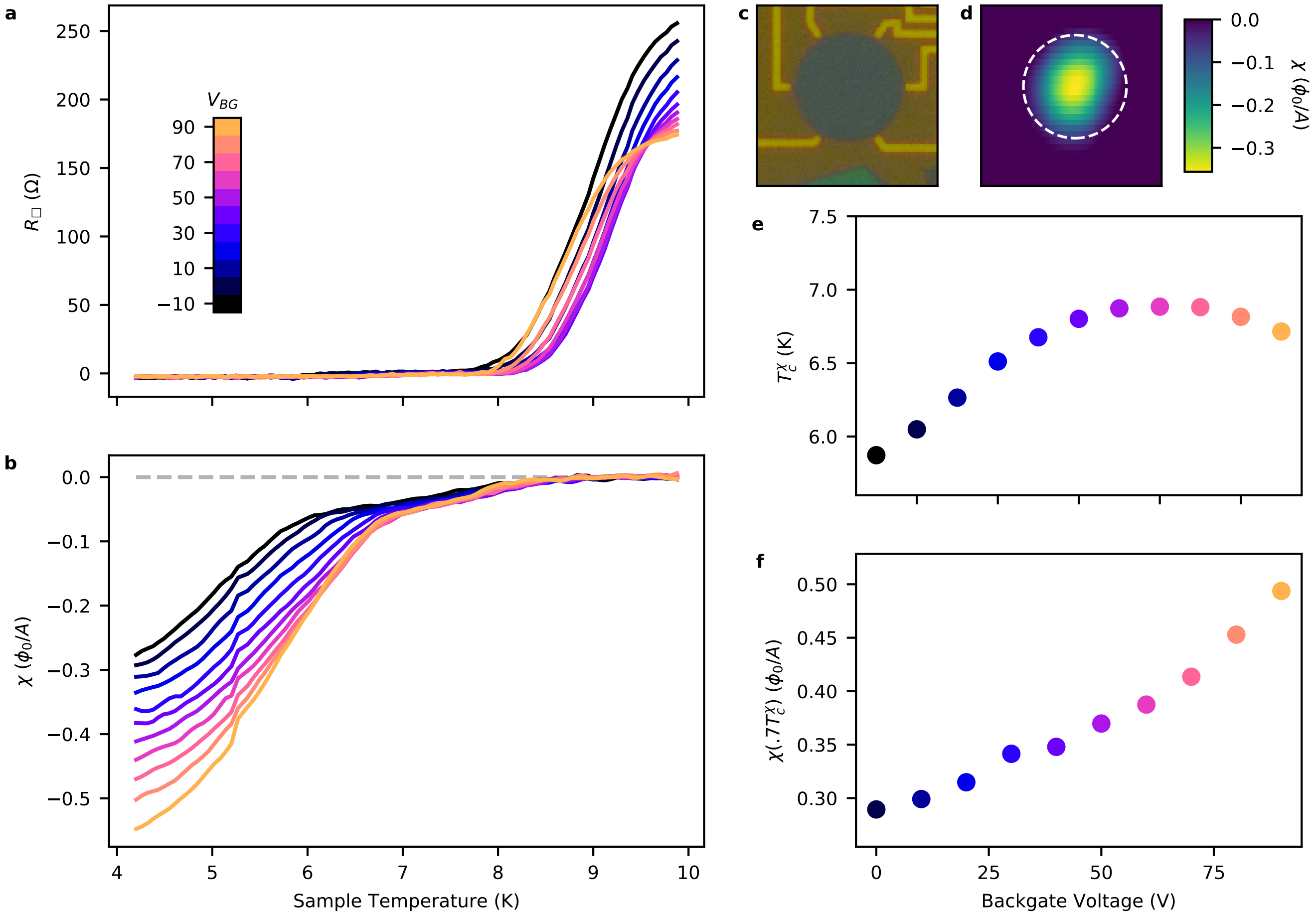

Supplementary Figure 3: Data from additional device C. a, Sheet resistance of device C versus temperature. Different curves correspond to different backgate voltages, as indicated by the inset color bar. **b**, Magnetic susceptibility of device C. Note the initial, slow rise in diamagnetism beginning around 8 K, and then rapid increase below 6 K. Line colors correspond to the color bar in panel a. **c**, Optical image of 14.2 μm MoS_2 disk before application of ionic gel. **d**, 4 Kelvin susceptibility map of sample. White dashed outline corresponds to 14.2 μm diameter of superconducting disk. **e**, Temperature of strong upturn in diamagnetism, T_c^χ, versus backgate. **f**, Strength of magnetic susceptibility at 70% of T_c^χ.

suppress the superfluid response initially, and that the superfluid response rise sharply at a vortex-anti-vortex binding transition. However, other explanations such as the presence of small length scale non-uniformities may also yield similar behavior. In addition, during measurements on device C, the external magnet was not included in the experimental setup yet. Therefore, a small out-of-plane magnetic field may have been present, which we estimate to be at most 14 μT or 1 flux quanta through the device. This may also modify the response of the device close to the transition.

Supplementary Methods 1: Determining the SQUID height and geometry

We detect the superfluid response as a change in mutual inductance between the pickup loop and field coil. The magnitude of this change depends on the superfluid stiffness of the superconducting device, on the height of the SQUID above the superconductor, and the point spread function (PSF) of the SQUID. We imaged Pearl vortices in a quasi-infinite 20 nm thick Nb film to characterize the PSF. To determine the height, our SQUID is placed on a brass cantilever which forms one side of a parallel plate capacitor. By measuring this capacitance, we can detect the touchdown of the SQUID on the sample. However, even at touchdown the height of the pickup loop above the sample is unknown due to a height offset resulting from the geometry of the SQUID chip and the unknown thickness of the frozen ionic liquid. We can estimate the geometric height from the angle between the SQUID and the sample and the distance from the front corner of the SQUID chip to the SQUID pickup loop. To estimate the thickness of the frozen ionic liquid, we image the magnetic field produced by the current in a lithographically well-defined structure. To first verify this approach, we fabricated 40 μm inner diameter, 42 μm outer diameter rings on test sample without ionic liquid. We then scanned the SQUID over the ring structure and located the geometric center of the ring. Then, we varied the height of the SQUID while recording the mutual inductance between the SQUID and ring. To produce a model to fit our data, we calculated the field of a finite width current carrying annulus, and convolved this field with the previously determined PSF. To compare this model to the data, the height of the SQUID in physical volts on the piezoelectric bender must be converted into height above the sample in meters. We introduce three parameters: the offset height z_0 of the SQUID when we detect touchdown, the derivative of height with respect to bender voltage m, and an effective scaling α of the PSF which allows the sensitivity of the SQUID to vary slightly from 1. This last parameter corrects for slight variations of the SQUID to sample angle between samples, which changes the effective area of the SQUID. If the angle, and therefore sensitivity, of the SQUID remains unchanged between the Nb film and the ring sample, we expect $\alpha = 1$. In the case of the current carrying ring on the test sample, we fix z_0 by geometry and fit m and α. We find $\alpha = .99 \pm .02$ where the uncertainty is the fitting uncertainty. There are two errors here, the fitting uncertainty (2%) and the difference in sensitivity from 1 (1%). We generously estimate our overall error as 5% in the signal magnitude. In the case of device B, we fabricated identical rings and performed the

same measurements as on the test sample. We fix m and α to the values found from the test sample, and fit z_0. We propagate the error in α by numerically computing derivative of the fit z_0 with respect to α. We finally obtain that the height of the SQUID in device B was 2.5 ± 1.0 μm. This may be compared to the geometric height of the SQUID if the front corner of the chip is in firm contact, which we estimate to be 0.8 μm. Thus, we find that the ionic liquid is 1.7 ± 1.0 microns thick. Device A, which was measured before device B, also had current-carrying rings, but they were too small to allow meaningful measurements. Therefore, since both devices were spin coated with ionic liquid in identical processes, and the SQUID was approached using the same procedure, we assume the SQUID height was the same in both measurements. Conversely, device C was measured earlier, with a different batch of ionic liquid, and therefore may have a different height.

Supplementary Methods 2: Modeling M_{geo}

To relate ρ_s and χ measured at a fixed height, we need to model the screening currents flowing in the sample in response to the field applied by the field coil. From this current density, we can calculate the magnetic field produced by the sample and the amount of flux coupled into the SQUID pickup loop. To model the screening currents, we adapt a numerical model employed in the literature for two-coil measurements of superconductors to our SQUID and field coil geometry [1]. The disk-shaped sample is modeled as a finite number N of infinitely thin superconducting rings with the radius of the ith ring given by $\mathbb{R}_i$ reflecting the circular symmetry of the problem. We approximate the field coil as circularly symmetric generating a magnetic field in the sample plane that is determined by a vector potential $\mathbb{A}(t) = \mathbb{A}_0 e^{i\omega t}$. Here the boldface indicates a vector indexing over the rings. The vector potential may vary in the radial and out-of-plane directions. Using Ohm's law with a complex conductivity, the current in the superconducting disk can be written as:

$$\mathbb{J} = -(\sigma_1^{2D} + i\sigma_2^{2D}) i\omega \left(\frac{1}{2\pi\mathbb{R}} M w \mathbb{J} + \mathbb{A}_0 \right) \tag{1}$$

$\mathbb{J}$ is a vector indexing over the rings with $\mathbb{J}_i$ the current in i^{th} ring. $M_{i,j}$ is the mutual inductance between the ith and j^{th} rings. The term $M\mathbb{J}$ takes the field created by the superconductor itself into account. The division by $\mathbb{R}$ is performed element-wise onto the result of $M\mathbb{J}$. Typically in a superconductor, $\sigma_1^{2D} = 0$ and $\sigma_2^{2D} = -2/(\omega\mu_0\Lambda) = -4k_B e^2 \rho_s/(\hbar^2\omega)$. Because $\mathbb{A}_0$ is the applied vector potential, $\mathbb{J}$ can be computed in principle by a matrix inversion. We compute the coefficients of M for $i \neq j$ by using a closed form in terms of elliptic functions for the vector potential of a current carrying ring [2]. For $M_{i,i}$ we use the results from Ref. [3]. To calculate $\mathbb{A}_0$, the field coil is modeled as carrying a circular current with a finite radial width and zero thickness in the z-direction. Then, the magnetic field everywhere can be computed using the resulting $\mathbb{J}$. Finally, we convolve the PSF discussed in the previous section with this magnetic field to compute the flux through the SQUID. We repeat this calculation as a function of

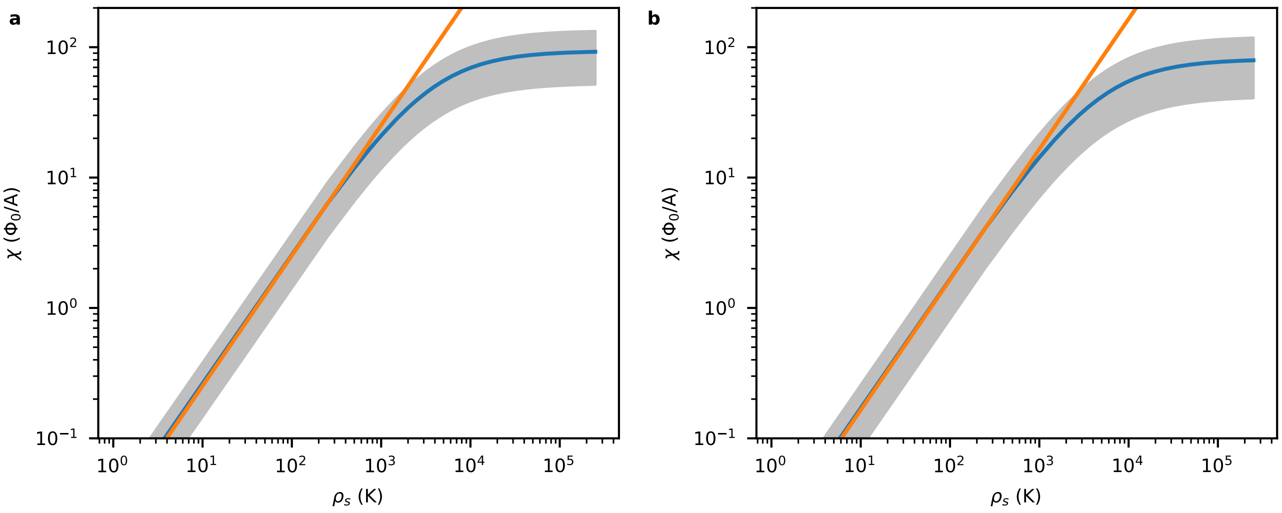

Supplementary Figure 4: Relation between χ and ρ_s. a, Relation between the superfluid stiffness and χ of device A, the magnetic response as measured by the SQUID. Blue line corresponds to the best estimate of the height, gray band indicates the uncertainty. The orange line is the linear fit to the weak screening limit. **b,** the same as a, but for device B.

ρ_s to create a conversion table between a measured value of χ and the corresponding value of ρ_s. To estimate the uncertainty coming from uncertainty in the SQUID height, we also perform this calculation for different heights, and apply the uncertainty in the height previously found to predict the uncertainty in stiffness reported in the main text.

Supplementary Discussion 1: Magnitude of measured superfluid response

In three dimensions, a superconductor reaches the ideal Meissner limit (internally $B = 0$) once its dimensions are much larger than the magnetic screening length. In such a limit all the magnetic screening currents flow on the surface. Similarly, if a two-dimensional superconducting disk is placed in a uniform field, and Λ is much smaller than the radius, screening currents will primarily flow at the edges. In the other limit, where Λ is much larger than the radius, the superconductor screens so little field that the current is proportional to the vector potential of the applied field, with appropriate gauge choice, and increases linearly with increasing distance from the center. To compare our data to these two limits, in Supplementary Fig. 4 we plot the relationship of χ and ρ_s at fixed height, utilizing the calculation from the previous Supplementary Methods section. In the low stiffness regime, χ and ρ_s are related linearly, as the distribution of $\vec{J}$ does not change, only its overall magnitude. The full range of χ we report in this work falls within this regime. As the stiffness passes 1000 K, the current begins to move to the edges of the device, leading to a more complex relationship between χ and ρ_s. Finally, χ saturates as the total circulating current in the device saturates, and its distance from the edge of the device becomes a negligible fraction of the SQUID dimensions. The stiffness we infer from our measurements is approximately two orders of magnitude lower than the value of the

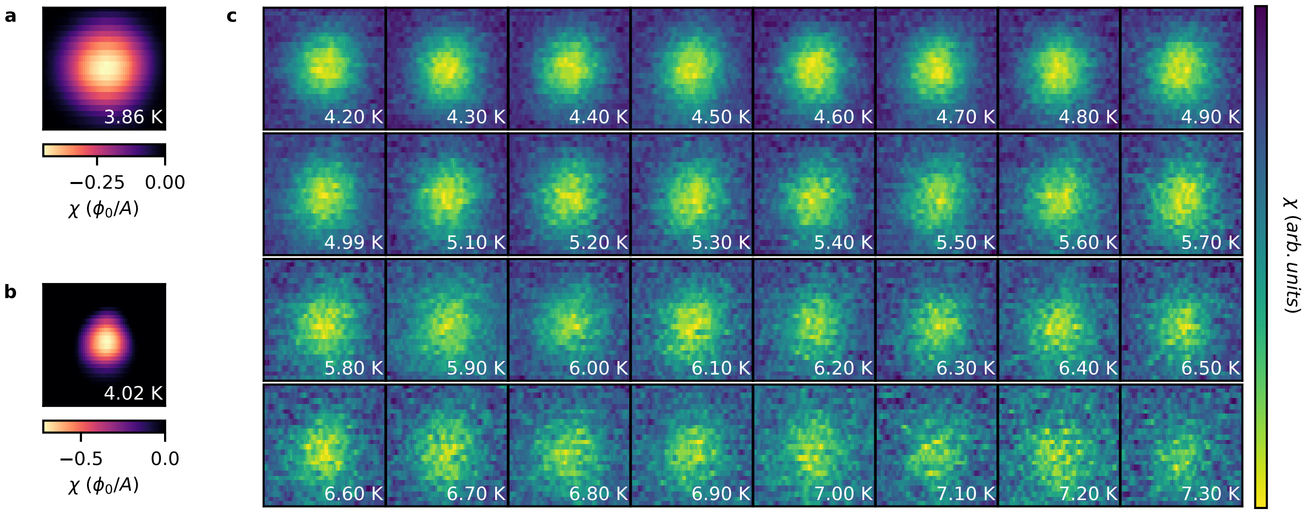

Supplementary Figure 5: Susceptibility images of devices. a, Spatial map of χ of device A. Field of view is approximately 22.5x22.5 μm. **b,** Same as a, but for device B. **c,** Spatial map of χ of device C, as a function of temperature. Data from each temperature are individually normalized as data at high temperature are much weaker. Field of view in each map is approximately 22.5x22.5 μm

onset of this saturation regime.

We can compare the strength of the screening current that are excited during our measurements to the critical currents determined by electrical transport measurements. In a transport experiment, a current is applied to a device, and the resulting voltage is read out using separate contacts. In our measurement, a circulating current is induced magnetically, and read out magnetically. A meaningful scale for both measurements is the critical current of the superconductor. Based on the previous Supplementary Methods section and the stiffness reported in the main text, we can estimate the current density in the device. Since the device is in weak screening, the largest currents will occur near the edge, and we estimate these to be .19 A/m and .35 A/m in devices A and B respectively. Critical current measurements reported in the literature suggest J_c on the of order 10 A/m [4, 5]. Therefore, only a small fraction of the critical current density flows in our measurements.

Supplementary Discussion 2: Effect of a normal conducting region along the circumference of a disk-shaped device

Our devices exhibit a backgate-dependent finite resistance even when we detect a superfluid response. One possible origin of a finite resistance in a superconducting device is a non-superconducting line defect dividing the contacts used to perform the transport measurement. However, we can exclude this type of defect as it would have a strong signature in images of χ as the SQUID is scanned over the sample, even if the line defect was much narrower than our spatial resolution [6]. In Supplementary Figs. 5a and b, we show maps of χ for device A and B, respectively. If a line defect divided the superconducting region in two, we would expect to see two separate peaks, which we do not

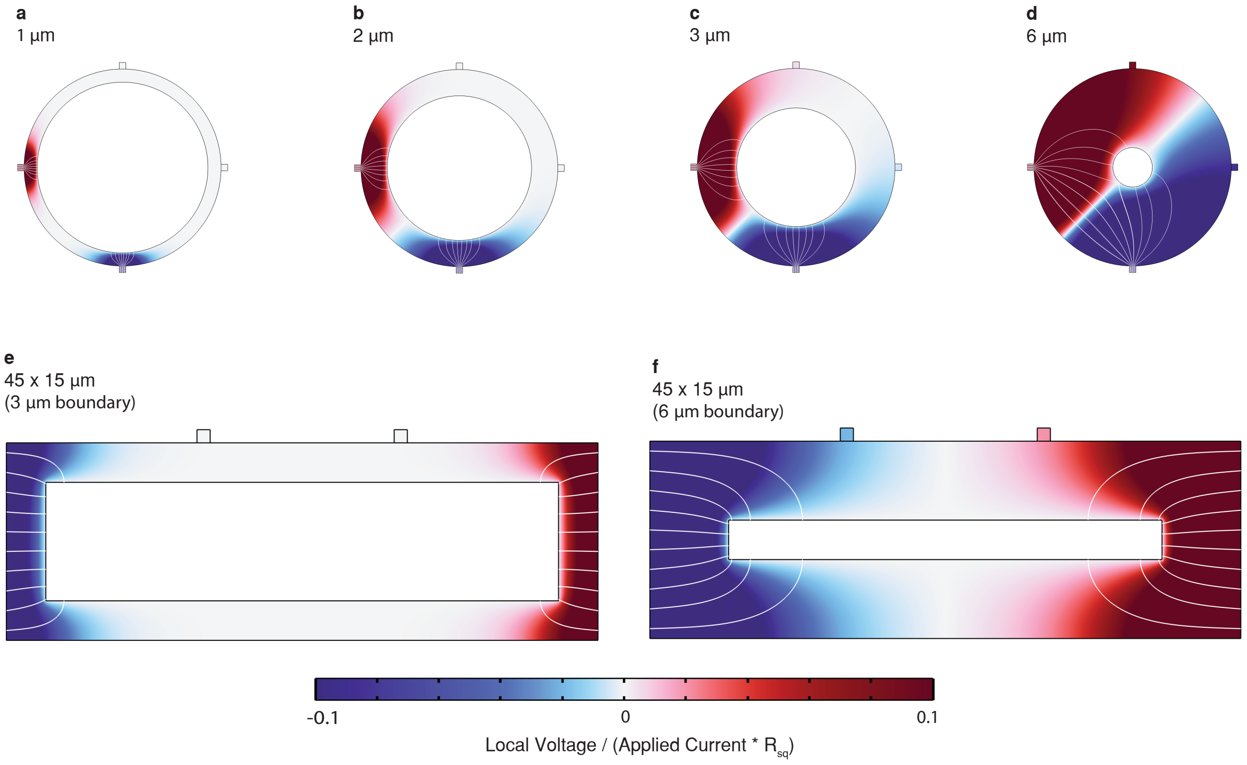

Supplementary Figure 6: Simulation of voltage in mixed superconductor/normal structures. a, 15 μm disk with a 1 μm non-superconducting boundary. Current is not simulated in superconducting region. Color scale is dimensionless, local voltage per unit current applied and unit sheet resistance. White streamlines show current density **b,** Same as a, but for a 2 μm boundary. **c,** Same as a, but for a 3 μm boundary. **d,** Same as a, but for a 6 μm boundary. **e,** Same as a, but for a 45 μm long, 15 μm wide Hall bar with a 3 μm boundary. **f,** Same as e, but for a 6 μm boundary.

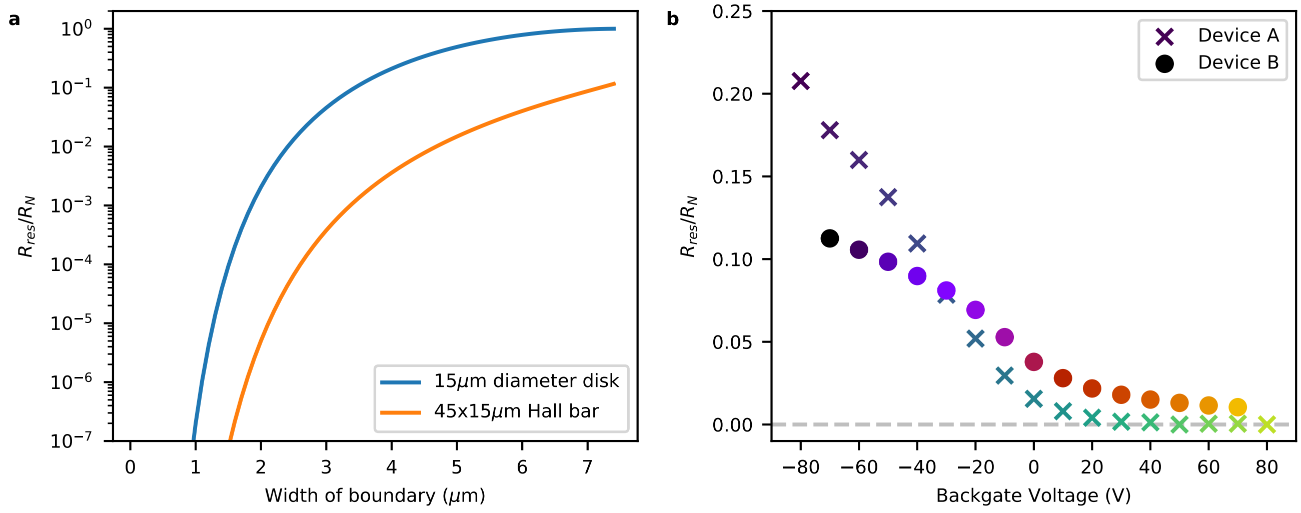

Supplementary Figure 7: Residual resistance ratio. a, Simulated ratio of the normal resistance of the device to the observed resistance in the numerical model, as a function of the radius of the superconducting region in the center. **b,** Experimentally measured ratio of the residual resistance to the normal resistance in device A and B as a function of the backgate voltage.

observe. In Supplementary Fig. 5c, we show spatial maps of χ for device C, as a function of temperature. The data show the approximately circular region of diamagnetic response disappearing while maintaining its shape, as opposed to breaking up into smaller regions. However, there are some non-uniformities that our imaging is less sensitive to. The apparent diameter of the diamagnetic response depends strongly on the height of the SQUID above the sample, which is much less well controlled during imaging than during measurements elsewhere in this paper in which the SQUID stays in a fixed position. Therefore, we cannot determine if a normal conducting periphery is present in our device.

Here, we explore if the presence of such a normal conducting ring around the perimeter of the disk-shaped devices can explain the observed finite resistance. Such a ring may form due to a combination of a non-uniform carrier density profile and the dependence of the critical temperature (and presence of superconductivity) on density. A non-uniform density profile may be present in our devices for several reasons: The backgate fringing fields can cause non-uniform density; this effect is confined to a few oxide thicknesses (300 nm) away from the edge of the device. In addition, ionic liquid gating of our devices is a complicated process, especially since the gate is set near the freezing temperature of the ionic liquid. If the accumulation dynamics cause a non-uniform distribution of ions above the device, in particular a lower density of ions close to the device boundary, this would give rise to significantly lower carrier density closer to the edge of the device.

In the following, we carry out simulations to estimate how wide a normal conducting ring is needed to explain the observed finite resistance. This width does depend on the assumed resistivity. We solve the following model system:

a superconducting disk of radius R_{SC} inside a normal metal ring of outside radius R. Electrical contact is made to the disk at 4 points, equally spaced around the outside radius R. Two adjacent contacts are used as the source and drain contacts, and the remaining two contacts are used as the voltage probes. We assume a conductor with zero resistance for the superconducting region, but ignore effects from the magnetic fields produced by the current density, which should be negligible given the long Pearl length of the superconducting samples. By symmetry, the superconductor must be at fixed potential halfway between that of the source and drain. Further, by virtue of the infinite conductivity of the superconductor, it must have this potential everywhere. Therefore, we can find the local potential in the normal conducting, annular region by applying a potential between the source and drain contacts and treating the boundary with the superconducting region as an equipotential line. This approach avoids instabilities associated with the large conductivity of the superconducting region when solving for the local potential numerically. Using COMSOL multi-physics, we then solve for the current flowing through the ring, and compute the corresponding resistance of the device measured in the four-point geometry. The voltage at which the superconductor is held is checked by ensuring that no net current flows in or out of the superconducting region. Supplementary Fig.6a-d show simulations in which the current is applied at the left contact, and drained at the bottom contact for different widths of the normal conducting region. The local potential is proportional to the applied current and the sheet resistance of the film. Therefore, we plot the local potential voltage divided by the product of the applied current and the sheet resistance, which is dimensionless. The color scale is chosen to highlight the potential near the voltage probes at the top and right sides of the device. From the simulations, we can extract the ratio of the residual resistance R_{res} to the normal resistance R_N. This ratio is shown in Supplementary Fig. 7a as a function of the width of the boundary region while assuming a total diameter of 7.5 μm for the disk. When the radius of the superconducting region is reduced to .25 μm, the ratio becomes 1, as the device is nearly entirely non-superconducting. Conversely, as the radius approaches 7.5 μm, the lithographic radius of the disk, the ratio goes to zero. In our devices, we find ratios of the residual resistance to the normal resistance as large as .21. We plot this ratio as a function of backgate voltage in Supplementary Fig. 7b . This would require an unrealistically large width of the normal ring, if its resistivity is assumed to be equal to the resistivity suggested by the normal state resistance. However, the resistivity close to the edge may be higher. Interestingly, the residual resistances observed in device A and B display a pronounced asymmetry with respect to $V_{\mathrm{BG}} = 0$ (see Supplementary Fig. 7b). In addition, device C shows almost vanishing resistance below T_c.

Finally, we note that the same width normal region along the device boundary has a much more pronounced effect in disk-shaped devices than it has in a traditional Hall bar geometry. In particular, for high-aspect ratio Hall bars with voltage probes far from the source and drain, the residual resistance will be comparatively small because the majority of the current will enter the superconductor before the positions where the voltage is measured. In Supplementary Figs. 6e,f, we show calculations similar to those applied to the disks for a 45x15 μm Hall bar. In

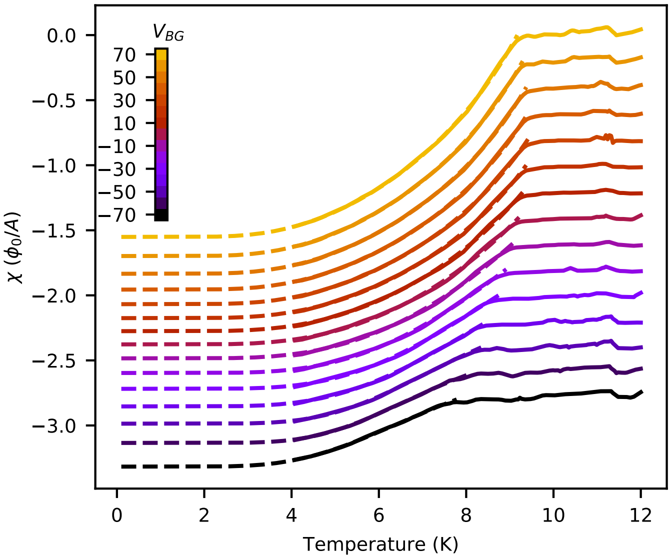

Supplementary Figure 8: Full BCS fits to Device B. Full fittings of the phenomenological model to the data from device B. Traces are vertically offset for clarity.

Supplementary Fig. 7a, we show the dependence of the residual resistance ratio on the width of the normal boundary for the Hall bar geometry shown in Supplementary Fig. 6e,f. This demonstrates that the residual resistance ratio in a Hall bar can be more than an order of magnitude lower than that for the disk geometry for the same boundary width, though details depend on the position of the voltage probes and size of the hall bar. This is because nearly all the transport current flows through the narrow superconducting strip in the central region. This demonstrates that it is easier to detect superconductivity in a Hall bar geometry compared to a disk geometry when a large periphery is non-superconducting. Despite circular devices not being an ideal geometry for transport, we utilize them to simplify the modeling of the SQUID data. We note that R_{res}/R_N of the Hall bar in Supplementary Fig. 7a does not approach 1 as the width of the boundary goes to 7.5 μm in a 15 μm wide Hall geometry. This is because the limiting behavior of the model is a superconducting line of arbitrarily narrow width which still reduces R_{res} below R_N. Conversely, in the disk geometry, the superconducting region shrinks to a point as the boundary width goes to 7.5 μm avoiding this artifact in the simulation.

Supplementary Discussion 3: Bounding the low temperature stiffness

In Fig. 3g,h of the main text, we report the inverse stiffness at low temperature versus sheet resistance. We compute the low temperature stiffness by fitting the phenomenological model in Eq. 16 from Ref. [7], and constrain $\alpha = 1$ and $\Delta/T_c = 1.76$. Here, we show those fits, and place additional bounds on the possible low-temperature superfluid stiffness. Generally, in both conventional and unconventional superconductors, $d\rho_s/dT$ is negative and steadily increases in absolute value as T increases [7], i.e. as we approach lower temperatures the curve tends to

flatten. Therefore, barring unusual order parameters like non-monotonic d-wave, we can place an upper bound on the magnitude of low temperature stiffness by extrapolating from 4 K to 0 K using a line with the slope of ρ_s at 4 K. Further, the value of $\rho_s(4\ \mathrm{K})$ provides a lower bound for $\rho_s(0\ \mathrm{K})$. In Supplementary Fig. 10a, we show the superfluid response curves for device A extrapolating from left to right with a constant line, the 4K slope, and the fit to T = 0K. Supplementary Fig. 10b shows the same for device B. Supplementary Figs. 10c,d show $\rho_s^{-1}(T = 0)$ estimated from the three methods shown in Supplementary Fig. 10a,b.

References

1. Turneaure, S. J., Pesetski, A. A. & Lemberger, T. R. Numerical modeling and experimental considerations for a two-coil apparatus to measure the complex conductivity of superconducting films. *Journal of Applied Physics* **83,** 4334–4343 (1998).
2. Garrett, M. W. Calculation of Fields, Forces, and Mutual Inductances of Current Systems by Elliptic Integrals. *Journal of Applied Physics* **34,** 2567–2573 (1963).
3. Gilchrist, J. & Brandt, E. H. Screening effect of Ohmic and superconducting planar thin films. *Physical Review B - Condensed Matter and Materials Physics* **54,** 3530–3544 (1996).
4. Costanzo, D., Jo, S., Berger, H. & Morpurgo, A. F. Gate-induced superconductivity in atomically thin MoS 2 crystals. *Nature Nanotechnology* **11,** 339–344. ISSN: 17483395. arXiv: 1512.03222 (2016).
5. Saito, Y., Itahashi, Y. M., Nojima, T. & Iwasa, Y. Dynamical vortex phase diagram of two-dimensional superconductivity in gated Mo S2. *Physical Review Materials* **4,** 1–10. ISSN: 24759953 (2020).
6. Kogan, V. G. & Kirtley, J. R. Meissner response of superconductors with inhomogeneous penetration depths. *Physical Review B - Condensed Matter and Materials Physics* **83,** 1–9 (2011).
7. Prozorov, R. & Giannetta, R. W. Magnetic penetration depth in unconventional superconductors. *Superconductor Science and Technology* **19** (2006).

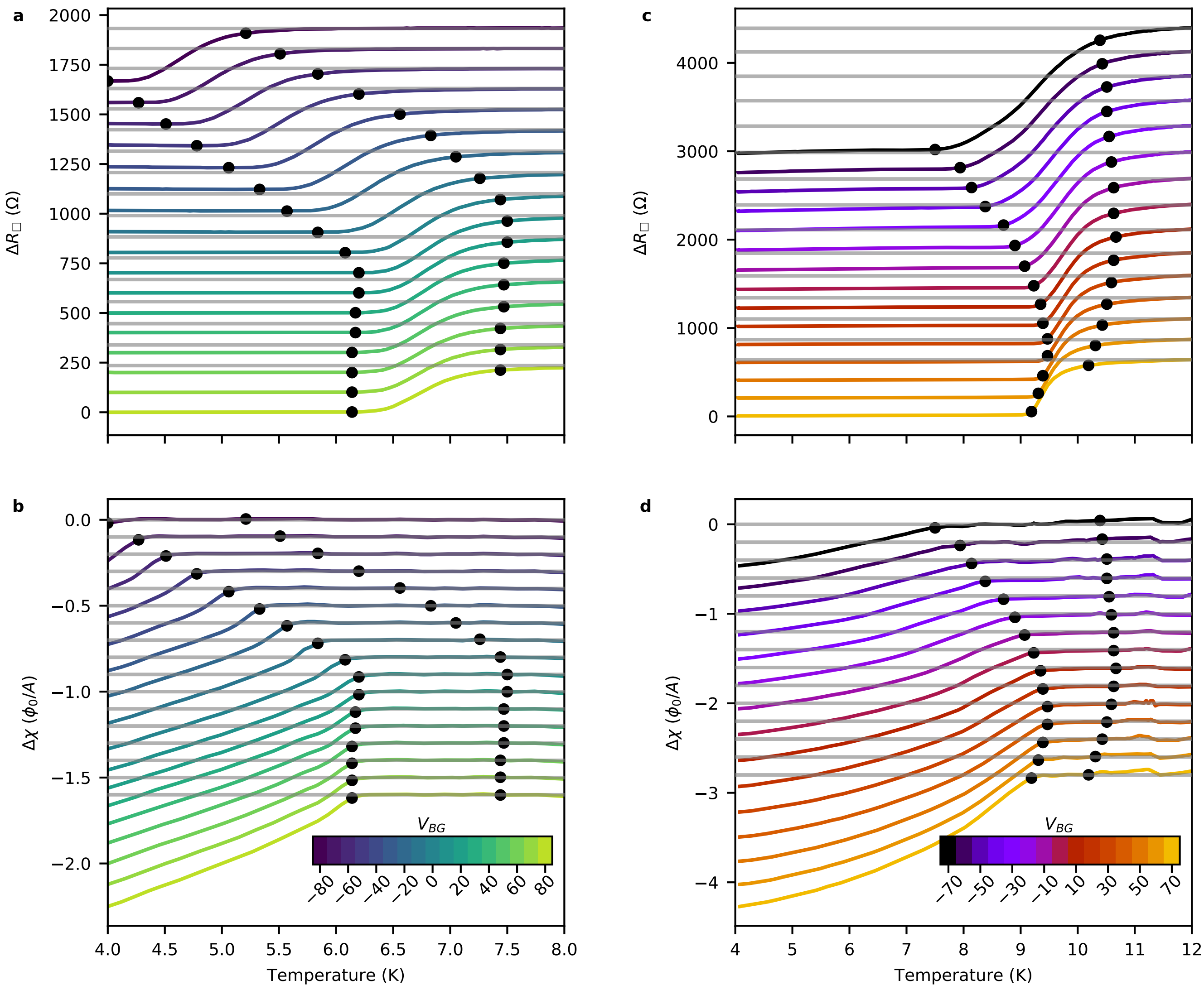

Supplementary Figure 9: $R_\square$ **and** χ **for device A and B with** T_c^χ **and** T_c^R **indicated. a**, Resistance data from Fig. 2a in the main text, offset in the vertical direction. Gray lines indicate 8 K resistance. Black circles at higher temperature are T_c^R, those at lower temperature are T_c^χ. **b**, Magnetic response curves from Fig. 2b in the main text, offset in the vertical direction. Gray lines indicate $\chi = 0$. Black dots the same as in a. **c**, Same as a, but for device B. **d**, same as b, but for device B.

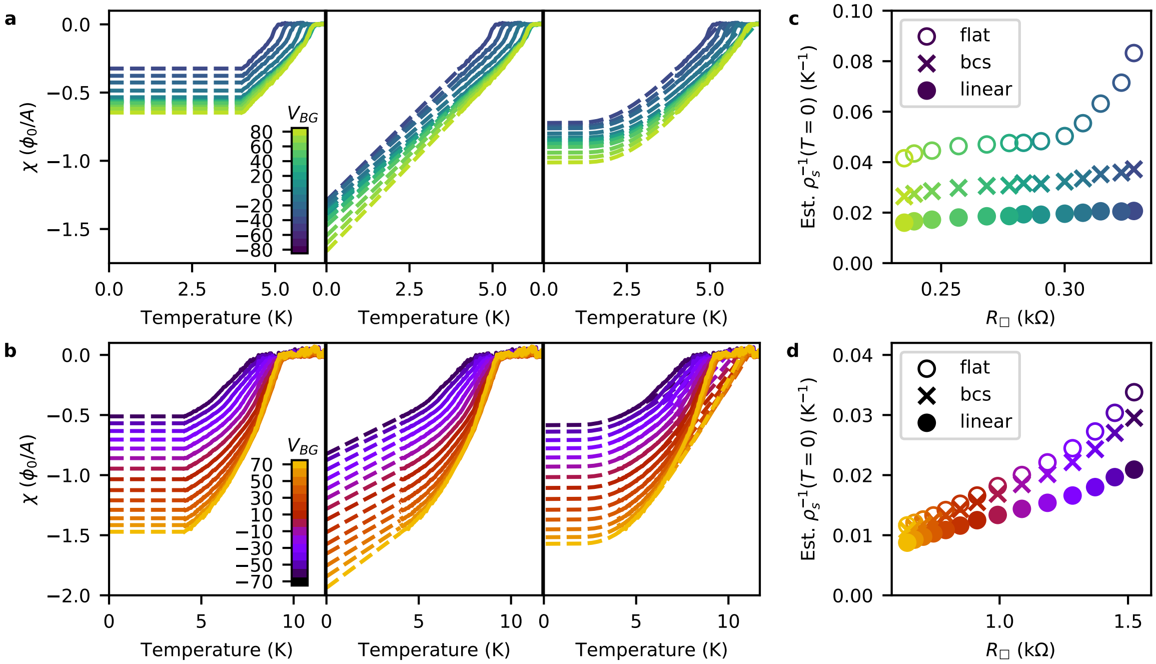

Supplementary Figure 10: Placing bounds on low temperature superfluid stiffness, $\rho_s(T = 0)$**. a**, Three methods of extrapolating χ in device A to zero temperature. From left to right: the flat method which assumes no more growth in χ occurs, the linear method, which linearly extrapolates from the slope at the lowest temperature, and the fitting of the BCS model with $\alpha = 1$ and $\Delta/T_c = 1.76$ to the data below 90% of T_c (the method used in Fig. 3c of the main text). **b,** Three methods of extrapolating χ in device B to zero temperature. From left to right: the flat method which assumes no more growth in χ occurs, the linear method, which linearly extrapolates from the slope at the base temperature, and the fitting of the BCS model with $\alpha = 1$ and $\Delta/T_c = 1.76$ to the data below 55% of T_c (the method used in Fig. 3d of the main text). **c,** Calculated device A zero temperature stiffness from the three methods. Note the poor agreement between the flat method and the other two methods for the highest resistance, most negative backgate curves. This is due to the clear proximity of T_c to the base temperature in these data, leading to very little stiffness at base temperature and hence a large inverse superfluid stiffness. **d,** Calculated device B zero temperature superfluid stiffness from the three methods. Note the quite good agreement between the three methods, better than the height error reported in the main text.
